# Analysis of Prussian carp (*Carassius gibelio* B.) oocytes under the influence of Roundup^®^ herbicide

**DOI:** 10.5114/bta.2023.132771

**Published:** 2023-12-21

**Authors:** Klaudia Kwiatkowska

**Affiliations:** Faculty of Environmental Engineering and Energy, Cracow University of Technology, Kraków, Poland

**Keywords:** glyphosate, ecotoxicology, herbicides, environmental protection

## Abstract

The aim of this study was to investigate the effect of Roundup^®^ herbicide on the maturation of Prussian carp oocytes under laboratory conditions. The Prussian carp is currently one of the most common fish species in Polish freshwater ichthyofauna. For the investigation, oocytes from five sexually mature female Prussian carp were used, segmented into three groups, and incubated for 24 h in Cortland’s saline, treated with varied concentrations of the herbicide Roundup^®^ (0 ng – control, R1 – 10 ng/ml, and R2 – 100 ng/ml). Subsequent to this period, assays were performed using the prepared plates to determine the level of 17α,20β-dihydroxyprogesterone (17α,20β-P) utilizing the standard ELISA technique. In determining the 17α,20β-P via ELISA, the medium was extracted from each tested oocyte group. Oocyte maturity was assessed through preservation in serra fluid, and, to categorize the maturity stage of the oocytes utilizing a four-point scale – contingent upon the nucleus’s position – the formerly preserved oocytes were dehydrated and subsequently analyzed. A contrast was noted in the percentage of oocytes at varied stages between the control group and the experimental groups. Specifically, a higher concentration of Roundup^®^ (100 ng/ml) accelerated to expedite the initial migration of the nucleus in oocytes. In conclusion, the obtained results show the adverse effect of Roundup^®^ on hormonal regulation and maturation in Prussian carp oocytes.

## Introduction

The development of agriculture and the increased demand for arable lands, driven by the constantly growing human population, often culminate in excessive chemicalization. A variety of products, classified as agrochemicals, find widespread use in this context. While their broad application aims to enhance production efficiency, it has several negative side effects. Improperly managed chemicalization not only induces soil degradation but also plays a devastating role in the eutrophication of water reservoirs (Malkan, 2020). The escalating use of agrochemicals poses significant challenges to environmental protection, especially in countries where there are no strictly defined organizational and legal aspects. Consequently, the scientific literature is replete with debates regarding the advisability of using chemicalization in agriculture. The justification for its use can ideally be weighed by juxtaposing the economic benefits to humanity against the collateral damage inflicted upon the natural environment. An appropriate scale of values and priorities should be developed, so that interests, consisting of the production of the necessary, high-quality agricultural products, coexist with the rational use of ecosystems (Gałązka and Głodowska, 2018).

The application of glyphosate, one of the most widely used herbicides in the world, is full of strong repercussions. Notable uproar enveloped Europe following the European Commission’s decision to renew glyphosate licenses (Commission Implementing Regulation (EU) No 844/2012), given that farmers have relied on glyphosate herbicides for over forty years (Myers et al., 2016). Findings from the Agricultural Health Survey (AHS) concerning glyphosate exposure – and the possibility of developing prostate cancer (relative risk, RR, 1.1; 95% CI, 0.9–1.3) and multiple myeloma (relative risk, RR, 1.1; 95% CI, 0.76–1.87) – ignited vibrant debates in 2015 following their publication. Consequently, the World Health Organization’s International Agency for Research on Cancer (IARC) concluded that glyphosate might be “potentially carcinogenic” (Baldi et al., 2015).

Glyphosate, the active ingredient in Roundup^®^ and certain other nonselective herbicides is formulated to destroy plants, with particular efficacy against weeds in crop fields (Papaioannidou, 2010). The success of this herbicide in decimating weeds is primarily based on the inhibition of a crucial enzyme in the biosynthesis of aromatic amino acids – specifically, shikimic acid – EPSPS synthetase (5-enolpyruvylshikimate-3-phosphate) (Gałązka and Głodowska, 2018). The National Pesticide Information Center (NPIC) indicates that half of the glyphosate in dead leaves decomposes within nine days and can remain in soil for up to 6 months (Henderson et al., 2010). Under favorable soil and weather conditions, plant protection products may enter surface waters, washing from agricultural lands where chemicalization was conducted (Stańczyk, 2020). Consequently, to mitigate side effects, governments have established limits on the usage and concentration of glyphosate-based pesticides in water reservoirs. For instance, the US permits 700 μg/l in tank water, Canada allows 280 μg/l in drinking water, while European regulations are more strict, permitting only 0.1 μg/l, thereby potentially offering more restrictive in terms of protecting aquatic biodiversity (Gonçalves et al., 2019). A study showed that concentrations of glyphosate below 0.1 μg/l present a low risk, while those above 1 μg/l – a level below the legal limit in some countries – pose a significant risk to aquatic organisms (Rehman et al., 2020). Aquatic ecosystems exhibit particular sensitivity to alterations prompted by human activity. Even minuscule concentrations of glyphosate pesticides, on the order of several ng/dm^3^, can disrupt numerous metabolic processes across various species of aquatic fauna and flora, including multiple species of fish, shrimp, algae, and even planktonic organisms (Hakeem et al., 2020). Such micropollutants depend not only on the concentration of the active compound but also on persistence, exposure time, and bio-accumulation capacity. Pesticides typically exhibit poor biodegradability, thereby enhancing their persistence in the environment (Davico et al., 2020). Hence, comprehending the risks posed by glyphosate-based herbicides is vital, not only for agricultural workers and food consumers but, most critically, in understanding their threats to the natural environment (Sylwestrzak et al., 2016).

In Europe, Prussian carp exist as a same-sex population, reproducing via gynogenesis. This mode of reproduction does not necessitate egg fertilization. A brief (a few minutes) contact with the sperm of other carp species suffices to activate egg development. Consequently, there is no crossing-over, which, under normal meiotic conditions, provides genetic variation; thus, the offspring of each gynogenetic female is a genotypically identical clone. This capability ensures that Prussian carp can reproduce both rapidly and efficiently (Kuhl et al., 2022).

Proper reproduction is contingent upon the maturation of the oocytes. In lower vertebrates, this is triggered by the maturation-inducing hormone (MIH), which interacts with receptors situated on the oocyte membrane, inducing the activation of the maturation-promoting factor in the oocyte cytoplasm. During maturation, oocytes undergo drastic morphological changes, correlated with the progression of the meiotic cycle and the breakdown of the nuclear envelope. Typically, the disintegration of the nuclear envelope is recognized as a hallmark of oocyte maturation (Zeng et al., 2017). Oocyte growth is facilitated by the accumulation of yolk proteins within their cytoplasm. Following the growth phase, the oocyte becomes primed for the next stage of oogenesis: the resumption of meiosis. This process, known as final oocyte maturation, takes place before ovulation and is a prerequisite for successful fertilization. It encompasses the breakdown of the embryonic vesicle (GVBD), chromosome condensation, the formation of the meiotic spindle, and the generation of the first polar body (Nagahaha and Yamashita, 2008). Furthermore, 17α,20β-dihydroxyprogesterone (17α,20β-P) is identified as a MIH (Sinhorin et al., 2014) and is also the most powerful steroid for inducing oocyte maturation in several fish species. The study presented herein was conducted to determine the effect of adding Roundup^®^ herbicide to the reproductive processes of Prussian carp.

## Materials and methods

### Animals

The research was conducted on five sexually mature female Prussian carp at the end of June 2020, during the full reproductive season. The fish were obtained from the Department’s Fisheries Experimental Station of Nutrition, Animal Biotechnology, and Fisheries in Mydlniki, Poland. The Prussian carp is the most prevalent species of fish in Poland’s freshwater ichthyofauna. Moreover, it is a gynogenetic fish (Kotusz, 2014). Its specific genetic basis of reproduction presents an intriguing research model for understanding the regulatory mechanism of oocyte maturation (Xie et al., 2001).

After being caught, the animals were weighed. Measuring body weight was necessary to determine the gonadosomatic index, which is an indicator of a fish’s seasonality and allows for the determination of ovary maturity.

### Analysis of Roundup^®^ influence on oocytes

The 0.5 ml of oocytes, obtained from the body cavity of the fish, were placed in a 24-well microplate along with the addition of Cortland’s saline culture medium, with the wells being topped up to a volume of 1 ml. Oocytes collected were divided into three groups: control group I (with Cortland's medium only), group II, and group III. For the test groups, in addition to the culture medium, two concentrations of Roundup^®^ herbicide – 10 ng/ml (R1) and 100 ng/ml (R2) – were supplemented. The prepared plates were incubated for 24 h at 21°C. Subsequently, oocytes were fixed with Serra fluid (a mixture containing 96% ethanol, formalin, and acetic acid). An equal volume (0.2 ml) of oocyte preservation fluid was introduced to the oocytes and mixed. Utilizing Serra fluid enables the dehydration process to expose the cell nucleus, a step that is essential for assessing the maturity of the oocytes (4-point scale). In this manner, 100 oocytes from each group were analyzed using a light microscope (Kamiński et al., 2020).

### Determination of 17α,20 β-dihydroxyprogesterone using ELISA

#### Hormone extraction

From each microwell, 100 μl of medium with oocytes were taken and transferred to Eppendorf tubes. Subsequently, 900 μl of dichloromethane was added, and the mixture was shaken dynamically. The prepared samples were then centrifuged for 5 min at 1500 RPM. The organic layer was transferred to a new test tube. The remaining aqueous layer was replenished by adding 900 μl of dichloromethane, followed by a repetition of the shaking and centrifugation processes. The organic layer was again collected and combined with the previous one. To evaporate the solvent, the samples were left on a lab bench at room temperature. After drying, 100 μl of ethanol was added to each sample.

#### Well coating

To each empty well of the microplate, 200 μl of carbonate buffer containing a monoclonal antibody (Monoclonal Anti Goat/Sheep IgG – SIGMA Chemical Co., USA) at a dilution of 1 : 5000 was added. The plate was then incubated at 4°C overnight. Subsequently, the microplate was washed three times with PBST buffer.

#### Blocking antigen – antibody complexes

To each well, 200 μl of casein solution (Kazeina [9000-71-9], POL-AURA, Poland) was added as a blocking agent. The microplate was then incubated at 37°C for 2 h. Following this, three washes in an Atlantis washer (Biochrom Asys, USA) with TBST (Tris-buffered saline, 0.1% Tween 20) buffer were performed. The filter paper was used for drying.

#### Incubation with antibody

To each well of the microplate, the following were added: 20 μl of each sample (in duplicates) or the appropriate standard 17α,20βP solution (BioVendor, Czech Republic) at concentrations of 0.1, 0.3, 1.3, 10, 30, 100, 300, 1000, and 3000 ng/ml; 50 μl of marker (hormone and peroxidase complex) (BioVendor, Czech Republic) at a dilution of 1 : 16000, and 50 μl of anti17α,20β-P antibody (BioVendor, Czech Republic) at a dilution of 1 : 20000. In addition, the substances used in this step were added omitting the samples (to observe the maximum binding – control samples), and the substrates used were assessed without considering the specific antibody (to generate nonspecific bindings). Thereafter, the microplate was covered with parafilm and incubated at 4°C for 16 h

#### Enzymatic decomposition of the substrate

The microplate was washed three times with PBST buffer and then dried using filter paper. Subsequently, 150 μl of TMB chromogen (Thermo Fisher, USA) was added to each well, and the plate was incubated in the dark for 30 min at room temperature.

#### Stopping the enzymatic reaction

To each well 50 μl of 2 M sulfuric acid was added and incubated at room temperature for 15 min.

#### Optical density measurement

Measurements were conducted at a wavelength of 450 nm using an EL 311 reader (BIO-TEK Instruments, Poland). Following the creation of a standard curve, the concentration of 17α,20β-dihydroxyprogesterone was determined.

#### Statistical analysis

The Mann-Whitney U test (STATISTICA, Poland) is a nonparametric test that allows for the comparison of groups and conditions without assuming that the values have a normal distribution. Therefore, all samples were analyzed by conducting measurements three times using the Mann-Whitney U test.

## Results

### Analysis of 17α,20 β-P level by ELISA

**Fig. 1:**
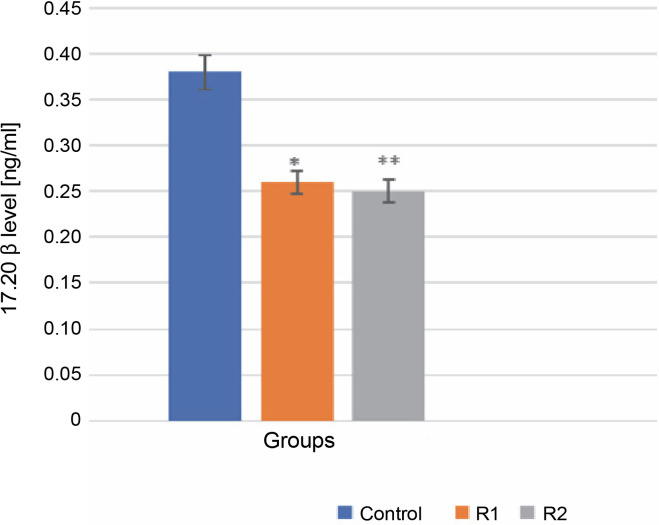
The effect of the herbicide Roundup^®^ on the secretion of 17α,20β-P from Prussian carp oocytes to incubation medium after 24 h; results for *n* = 5 are expressed as means ± error standard mean (SEM); statistically significant differences between the means marked * for *P* ≤ 0.05 and ** for *P* ≤ 0.001

The 17α,20β-dihydroxyprogesterone, also known as the MIH, is requisite for the meiotic maturation of oocytes. It is synthesized in the ovarian follicle from 17α-hydroxyprogesterone, a process catalyzed by the enzyme 20β-hydroxysteroid dehydrogenase. 17α,20β-DP acts through its receptor, 17α,20β-DPR, on the plasma membrane of oocytes (Ijiri et al., 2017). This synthesis necessitates the interaction between two layers of ovarian follicle cells: the thecal cell layer and the granular layer. Statistical analysis using the Mann-Whitney test showed significant differences in the level of 17α,20β-P released into the incubation medium in both the control and experimental groups (R1, R2). Specifically, the addition of Roundup^®^ herbicide at a concentration of 100 ng/ml precipitated a highly significant decrease in 17α,20β-P levels (reduction to 0.25 ng/ml), manifested by a diminished release of the hormone into the incubation medium. This may indicate that Roundup^®^ exerts an inhibitory effect on oocyte maturation (*P*-value ≤ 0.001, see Fig. 1).

To assess the oocyte maturity state, the oocytes were dehydrated to expose the cell nuclei. The maturity stages of Prussian carp oocytes are presented in Table 1.

**Table 1:** Maturity stages of Prussian carp oocytes based on the location of the nucleus

Stage	Description	Picture
I	Oocytes with centrally located nucleus	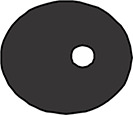
II	Early nuclear migration (nuclei shifted, but not more than halfway oocyte diameter)	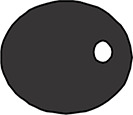
III	Late nucleus migration (nuclei shifted beyond half the oocyte diameter)	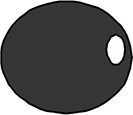
IV	Oocytes with a nucleus after GVBD (germinal vesicle breakdown)	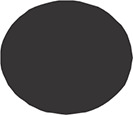

**Fig. 2:**
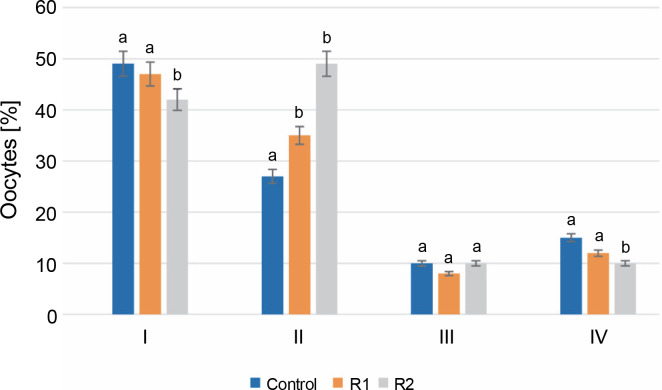
The assessment of the oocyte maturity state; the effect of the herbicide Roundup^®^ on the maturation of Prussian carp oocytes after 24 h *in vitro* incubation; results for *n* = 5 are expressed as means ± error standard mean (SEM); different letters determine statistically significant differences between the groups (*P* ≤ 0.05)

After 24 h of *in vitro* incubation with the herbicide, stage I oocytes constituted the largest percentage of all oocytes. In the control group, stage I oocytes accounted for 49% of the total analyzed cells, compared to 47% in R1 and 42% in R2. Analysis of the obtained results revealed statistically significant differences between the control group and the R1 and R2 groups. A similar trend was also observed for stage IV oocytes. The percentage of oocytes in stage IV, following germinal vesicle breakdown (GVBD), was the lowest and statistically significant in the R2 group compared to the other tested groups (15% for control, 11% for R1, and 10% for R2). Discrepancies in the percentages of oocytes at the stage of early cell nucleus migration (stage II) were noticeable in oocytes treated with the herbicide at both tested doses. At stage II, an increasing share of oocytes was observed along with an increasing dose of Roundup^®^. For the control, oocytes at this stage accounted for 26%, while herbicide treatment increased the percentage of stage II oocytes to 33% for R1 and 49% for R2. No statistically significant differences were found in the evaluation of stage III oocytes. The percentage of oocytes with a peripheral nucleus was similar in the groups treated with Roundup^®^ herbicide and in the control group (*P*-value ≤ 0.05, see Fig. 2).

## Discussion

Glyphosate-based herbicides (GBH) hold a significant stake in agriculture. In Poland, approximately one hundred herbicides containing glyphosate have been introduced into the market (Kwiatkowska et al., 2013). Studies on the effects of GBH in marine environments highlight toxic impacts not only on fish but also on crayfish, crabs, tadpoles, and frogs (Cuhra et al., 2016; Anifandis et al., 2018; Krimsky, 2021). Fish and amphibians, as vertebrate groups, reflect ecosystem function and structure (e.g., nutrient dynamics and cyclicality or zooplanktonic composition), especially in freshwater ecosystems (Marques et al., 2019).

Glyphosate is absorbed through both the gills and digestive tract of *Anguilla anguilla* fish following the ingestion of contaminated food. Once inside the body, it is absorbed and distributed via the bloodstream to all tissues and organs (Samsel and Seneffl, 2015). Research by Szarek et al. (2000) indicated that glyphosate elevated oxidative stress levels disrupted reproduction due to impairment of the estrogen pathway, and hindered the proper functioning of the brain in carp. Moreover, glyphosate impeded acetylcholinesterase activity at European eel synapses (Marques et al., 2019) and induced growth and fusion of gill plates in the gills of Neotropical fish, interfering with the gas exchange process’s efficiency (De Castro Sachi et al., 2021).

The compound might also be genotoxic to cells, a fact substantiated by histopathological tests in fish hepatocytes where vacuolization and cytoplasm degradation were observed (Samsel and Seneffl, 2015). A biochemical analysis of the liver, heart, and kidneys conducted on freshwater fish *Cyprinus Carpio* L. documented an increase in the activity of alkaline phosphatase and the activity of glutamine-oxaloacetate transaminase (Gonçalves et al., 2019). Furthermore, GBH reduced the activity of antioxidant enzymes (such as catalase, glutathione peroxidase, and superoxide dismutase) in fish species like rainbow trout (Hidalgo et al., 2002). Studies have demonstrated that the addition of Roundup^®^ decreased the activity of these antioxidant enzymes in the liver and brain in goldfish due to the augmented flux of superoxide radicals and H_2_O_2_. Free radicals inhibited the enzymatic activity and disrupted the normal antioxidant response in the liver and brain (Tokumoto et al., 2004).

Additionally, by inducing oxidative stress in the brain, the herbicide adversely affected *Prochilodus lineatus*. Results indicate that while the herbicide stimulates bio-transformation pathways, it reduces the activity of certain antioxidant enzymes and leads to lipid peroxidation (Modesto and Martinez, 2019). Glyphosate also influenced the immune system of rainbow trout, causing an increase in the production of proinflammatory cytokines and inflammation (Xie et al., 2005).

From an ecological point of view, the influence of GBH on reproductive processes is particularly important. The compounds contained in these agrochemicals negatively impacted hormonal activity and led to disturbances in the reproductive processes of teleost fish (Patino and Sullivan 2002). An *in vitro* study showed that low GBH concentrations (50, 300, and 1800 μg/l) killed *Astyanax lacustris* sperm, threatening reproduction and disrupting the natural persistence of the population (Samsel and Seneffl, 2015).

In this experimental work, analyses were performed to determine the effect of Roundup^®^ on the maturation of Prussian carp oocytes. To ascertain the influence of the glyphosate-based herbicide on the maturation of carp oocytes, the secretion of 17α,20β-dihydroxyprogesterone from oocytes to the incubation medium was analyzed. Two concentrations of Roundup^®^ herbicide were used: 10 and 100 ng/ml. The experimental doses for the experiments were selected based on soil analyses conducted in agricultural fields located near water reservoirs by the Department of Herbology and Technology Crops of the IUNG-PIB (Grygiel et al., 2012). In the experiment, the level of 17α,20β-dihydroxyprogesterone was notably lowered in the oocyte groups treated with Roundup^®^ herbicide. The observed change may account for the negative impact of the pesticide on the reproductive process of the Prussian carp. As oocytes grow, the epithelial cells produce testosterone, which is aromatized to estradiol-17β in the granule layer cells. When vitellogenic vesicles fully develop, 17α-hydroxyprogesterone is produced by the epithelial cells and then transformed into 17α,20β-P in the granular layer cells. The production of hydroxyprogesterone at the end of oogenesis regulates oocyte maturation and may negatively affect the final maturation processes and ovulation (Zohar et al., 2021).

Additionally, an analysis of the Prussian carp oocytes’ maturation process, based on nucleus migration during the 24-h incubation, was conducted. Statistically significant differences in the percentages of oocytes at stages I, II, and IV were observed between the studied groups. The differences observed between the control and experimental groups suggest that the addition of Roundup^®^ herbicide may enhance early cell nucleus migration. This is noteworthy since cytoplasmic maturity determines the oocyte’s ability to fertilize and to initiate and continue the embryo’s mitotic divisions (Opiela and Kątska-Książkiewicz, 2004). A higher concentration of the herbicide (100 ng/ml) may simultaneously interfere with migration towards the micropyle and the final maturation of oocytes, including GVBD, which can further disrupt the ovulation process. The addition of glyphosate at doses of 0.65 and 6.5 mg/l to *Danio rerio* oocytes disrupted ovarian maturation, observed as a reduction in ovarian follicle diameter, ultrastructural changes in oocytes, and disruption of the vitelline/chorion membrane (Davico et al., 2020). Delayed maturation of the ovaries was signaled by an increased number of primary follicles or a decrease in their size. Also observed were vacuolization in follicular cells and an increase in the perivitelline space. Additionally, changes in oocytes’ mitochondria, which manifested as a loss or expansion of cristae, were detected (Davico et al., 2020). Other studies on zebrafish found that glyphosate significantly inhibited oocyte maturation, thereby reducing fish fertility. GVBD was observed at 50 and 500 μM glyphosate concentrations (Zhang et al., 2019). Unfortunately, the reasons behind glyphosate’s inhibition of oocyte maturation in *D. rerio* are still unclear. It has been suggested that it could be caused by the inhibition of aromatase, an essential enzyme involved in estrogen synthesis (Maskey et al., 2019).

Oocyte maturation is a pivotal step in the reproductive process, and the reproduction of many species can be disrupted by numerous chemicals entering the environment. Endocrine disorders can be triggered by a variety of substances that may mimic hormones, act as receptor blockers, and/or serve as enzyme inhibitors (Maskey et al., 2019). Moreover, a myriad of side effects, including endocrine disorders, tissue damage, and gametogenesis dysfunctions, impact not only aquatic organisms. Despite this, the regulation of glyphosate and GBH remains insufficiently restrictive, especially in countries that have renewed authorizations for the next years. A pronounced need exists for further analysis, particularly concerning the effects of GBH, since glyphosate is seldom used in isolation. New studies can aid in standardizing glyphosate regulations and definitively resolving its toxicity status among regulatory agencies (Peillex and Pelletier, 2020).

## Conclusions

It has been demonstrated that Roundup^®^ herbicide diminishes the secretion of 17α,20β-dihydroxyprogesterone into the incubation medium from Prussian carp oocytes, potentially inhibiting the final processes of oocyte maturation. A higher concentration of Roundup^®^ (100 ng/ml) expedited the initial migration of the nucleus in oocytes while inhibiting subsequent stages of maturation and GVBD. Consequently, it can be posited that Roundup^®^ may interfere with the reproductive processes of female Prussian carp.

## Conflict of interest

The author declares no conflict of interest.

## References

[cit0001] Anifandis G., Amiridis G., Dafopoulos K., Daponte A., Dovolou E., Gavril E., Gorgogietas V., Kochpani E. (2018) The in vitro impact of the herbicide Roundup on human sperm motility and sperm mitochondria. Toxics 6: 2–10. 10.3390/toxics6010002 PMC5874775 29267194

[cit0002] Baldi I., Blair A., Calaf G.M., Egeghy P.P. (2017) Carcinogenicity of tetrachlorvinphos, parathion, malathion diazinon, and glyphosate. Lancet Oncol. 16: 488–491. 10.1016/S1470-2045(15)70134-8 25801782

[cit0003] Cuhra M., Bohn T., Cuhra P. (2016) Glyphosate: too much of a good thing. Front. Environ. Sci. 4: 1–14.

[cit0004] Davico C.E., Guimarães-Pereira A., Nezzi L. (2020) Reproductive toxicity of herbicide: impairments in ovarian follicles of model organism Danio rerio. Environ. Sci. Pollut. Res. 28: 15147–15159. 10.1007/s11356-020-11527-z 33226558

[cit0005] De Castro Sachi T., Bonomo M.M., Sakuragui M.M., Modena P.Z., Paulino M.G., Carlos R.M., Fernandes J.B., Fernandes M.N. (2021) Biochemical and morphological biomarker responses in the gills of a Neotropical fish exposed to a new flavonoid metal-insecticide. Ecotoxicol. Environ. Safety 208: 111459. 33069948 10.1016/j.ecoenv.2020.111459

[cit0006] Gałązka A., Głodowska M. (2018) Intensyfikacja rolnictwa a środowisko naturalne. Zesz. Probl. Post. Nauk Roln. 592: 3–13.

[cit0007] Gonçalves B., Cardoso P., Silva D. (2019) Ecotoxicology of glyphosate-based herbicides on aquatic environment. Biochem. Toxicol. Heavy Met. Nanomat. IntechOpen 10: 57–72.

[cit0008] Grygiel K., Sadowski J., Snopczyński T., Wysocki A (2012) Pozostałości herbicydów w płodach rolnych i glebie. J. Ecol. Health 16: 159–163.

[cit0009] Henderson A.M., Gervais J.A., Luukinen B., Buhl K., Stone D., Cross A., Jenkins J. (2010) *Glyphosate general fact sheet*. National Pesticide Information Center, Oregon State University Extension Services.

[cit0010] Hidalgo M.C., Exposito A., Palma J.M., de la Higuera M. (2002) Oxidative stress generated by dietary Zn-deficiency: studies in rainbow trout (Oncorhynchus mykiss). Inter. J. Biochem. Cell Biol. 34: 183–193. 10.1016/s1357-2725(01)00105-4 11809421

[cit0011] Ijiri S., Shibata Y., Takezawa N., Kazeto Y., Talatsuka N., Kato E., Ozaki Y., Yamauchi K. (2017) 17β-HSD Type 12-like is responsible for maturation-inducing hormone synthesis during oocyte maturation in Masu Salmon. Endocrinology 158: 627–639. 27967235 10.1210/en.2016-1349

[cit0012] Kamiński R., Sikorska J., Wolnicki J. (2020) Sztuczny rozród karasia pospolitego Carassius carassius (L.). Projekt: *Program doradztwa rybackiego „Rozradzanie, wylęgarnictwo, podchów ryb i zarybianie”*; etap II, akronim *DORADZTWO*.

[cit0013] Kotusz J. (2014) *Gatunki obce w faunie Polski – karaś srebrzysty*. Instytut Ochrony Przyrody PAN.

[cit0014] Krimsky S. (2021) Can glyphosate-based herbicides contribute to sustainable agriculture? Sustainability 13: 23–37.

[cit0015] Kuhl H., Du K., Schartl M., Kalous L., Stöck M., Dunja K. (2022) Equilibrated evolution of the mixed auto-/allopolyploid haplotype-resolved genome of the invasive hexaploid Prussian carp. Nature Commun. 13: 33–41. 35835759 10.1038/s41467-022-31515-w PMC9283417

[cit0016] Kwiatkowska M., Jarosiewicz P, Bukowska B. (2013) Glifosat i jego preparatytoksyczność, narażenie zawodowe i środowiskowe. Medycyna Pracy 64: 717–729. 24502134 10.13075/mp.5893.2013.0059

[cit0017] Malkan S. (2020) *Glyphosate fact sheet: cancer and other health concerns*. https://usrtk.org/pesticides/glyphosate-health-concerns

[cit0018] Marques A., Guilherme S., Gaivão I., Santosa MA., Pacheco M. (2019) Progression of DNA damage induced by a glyphosate-based herbicide in fish (Anguilla anguilla) upon exposure and post-exposure periods – insights into the mechanisms of genotoxicity and DNA repair. Compar. Biochem. Physiol. Toxicol. Pharmacol. 166: 126–133. 10.1016/j.cbpc.2014.07.009 25110831

[cit0019] Maskey E., Crotty H., Wooten T., Khan I.A. (2019) Disruption of oocyte maturation by selected environmental chemicals in zebrafish. Toxicol. Vitro 54: 123–129. 10.1016/j.tiv.2018.09.017 30266436

[cit0020] Modesto K.A., Martinez C.B.R. (2019) Roundup^®^ causes oxidative stress in liver and inhibits acetylcholinesterase in muscle and brain of the fish Prochilodus lineatus. Chemosphere 78: 294–299. 10.1016/j.chemosphere.2009.10.047 19910015

[cit0021] Myers J.P., Antoniou M.N., Blumberg B., Carroll L., Colborn T., Everett L.G., Hansen M., Landrigan P.J., Lanphear B.P. (2016) Concerns over use of glyphosate-based herbicides and risks associated with exposures: a consensus statement. Environ. Health 15: 1–19. 26883814 10.1186/s12940-016-0117-0 PMC4756530

[cit0022] Nagahama Y., Yamashita M. (2008) Regulation of oocyte maturation in fish. Develop. Growth Different. 50: 195–219. 10.1111/j.1440-169X.2008.01019.x 18482399

[cit0023] Opiela J., Kątska-Książkiewicz L. (2004) Charakterystyka zdolności rozwojowej oocytów ssaków w aspekcie zapłodnienia i rozwoju zarodkowego: I. Dojrzałość jądrowa i molekularne aspekty jej regulacji. Biotechnologia 66: 140–151.

[cit0024] Papaioannidou P. (2010) *8^th^ Southeast European Congress on Xenobiotic Metabolism and Toxicity, Xenoestrogens – Impact on Reproduction – XEMET*.

[cit0025] Patino R., Sullivan C.V. (2002) Ovarian follicle growth, maturation, and ovulation in teleost fish. Fish Physiol. Biochem. 26: 57–70.

[cit0026] Peillex C., Pelletier M. (2020) The impact and toxicity of glyphosate and glyphosate-based herbicides on health and immunity. J. Immunotoxicol. 17: 163–174. 32897110 10.1080/1547691X.2020.1804492

[cit0027] Rehman K., Bhat H.R.A., Qadri H. (2020) Concerns and threats of contamination on aquatic ecosystems. Bioremed. Biotech. 27: 1–26.

[cit0028] Samsel A., Seneff1 S. (2015) Glyphosate, pathways to modern diseases III: Manganese, neurological diseases, and associated pathologies. Surgic. Neurol. Inter. 6: 22–45. 10.4103/2152-7806.153876 PMC4392553 25883837

[cit0029] Sinhorin D.G., Sinhorin A.P., dos Santos Teixeira J.M., Lazarotto K.M., Hansen P.C. (2014) Effects of the acute exposition to glyphosate-based herbicide on oxidative stress parameters and antioxidant responses in a hybrid Amazon fish surubim (Pseudoplatystoma sp). Ecotoxicol. Environ. Safety 106: 181–187. 24840881 10.1016/j.ecoenv.2014.04.040

[cit0030] Stańczyk P. (2020), Glifosat nas truje! I jest wszędzie. Tyg. Spraw Obyw. 32: 46–49.

[cit0031] Sylwestrzak Z., Zgrundo A., Latała A. (2016) Wpływ Roundupu^®^ w tym glifosatu na okrzemkę Navicula perminuta (Grunow) hodowaną w mieszanej kulturze glonów i naturalnym zbiorowisku mikrofitobentosu. [in:] *Polscy doktorzy i doktoranci w rozwoju światowej myśli naukowej*. Ed. Weiland M., Woźniak M., Pilarz Ł.B., Drewniak M. Network Solutions Editors: 231–239.

[cit0032] Szarek J., Siwicki A., Andrzejewska E. (2000) Effects of the herbicide Roundup on the ultrastructural pattern of hepatocytes in carp (Cypinus carpio). Marine Enviro. Res. 50: 263–266. 10.1016/s0141-1136(00)00088-x 11460701

[cit0033] Tokumoto T., Tokumoto M., Horiguchi R. (2004) Diethylstilbestrol induces fish oocyte maturation. Proc. Nat. Acad. Sci. USA 101: 3686–3690. 14990787 10.1073/pnas.0400072101 PMC373523

[cit0034] Xie L., Irwin M.A., Thrippleton K. (2005) Evaluation of estrogenic activities of aquatic herbicides and surfactants using a rainbow trout vitellogenin assay. Toxicol. Sci. 87: 391–408. 16049272 10.1093/toxsci/kfi249

[cit0035] Zeng X., Li K., Yuan R., Gao H., Luo J., Liu F., Wu Y. (2018) Associated chromosome dynamics during meiotic prophase I. Front. Cell Develop. Biol. 5: 1–9. 10.3389/fcell.2017.00121 PMC5767173 29376050

[cit0036] Zhang J.W., Xu D.Q, Feng X. (2019) The toxic effects and possible mechanisms of glyphosate on mouse oocytes. Chemosphere 237: 124435. 31352102 10.1016/j.chemosphere.2019.124435

[cit0037] Zohar Y., Marvel M., Levavi-Sivan B. (2021) Gnrh2 maintains reproduction in fasting zebrafish through dynamic neuronal projection changes and regulation of gonadotropin synthesis, oogenesis, and reproductive behaviors. General Compar. Endocrinol. 11: 55–67. 10.1038/s41598-021-86018-3 PMC7987954 33758252

